# *Akkermansia muciniphila* helps in the recovery of lipopolysaccharide-fed mice with mild intestinal dysfunction

**DOI:** 10.3389/fmicb.2025.1523742

**Published:** 2025-03-12

**Authors:** Yue Hu, Jun Zhou, Xiaoqi Lin

**Affiliations:** ^1^Department of Physiology, Basic Medical College, Shenzhen University, Shenzhen, China; ^2^Shenzhen InnoStar Institute of Biomedical Safety Evaluation and Research Co., Ltd., Shenzhen, China

**Keywords:** *Akkermansia muciniphila*, lipopolysaccharide, gut microbiota, intestinal dysfunction, probiotics, inflammation, immune modulation

## Abstract

**Background:**

Mild intestinal dysfunction, linked to subtle yet significant health issues, can be induced by lipopolysaccharide (LPS), a Gram-negative bacterial component that disrupts gut function and triggers inflammation. *Akkermansia muciniphila* has shown promise as a probiotic for gut health due to its roles in mucin degradation and short-chain fatty acid production. This study explores the therapeutic effects of *Akkermansia muciniphila* on LPS-induced mild intestinal dysfunction in mice.

**Methods:**

Thirty-eight 6-week-old C57BL/6 mice were split into control (*n* = 19) and LPS-treated (*n* = 19) groups. LPS-treated mice received 300 μg/kg/day of LPS for 4 weeks, followed by *Akkermansia muciniphila* supplementation at 41 mg/kg/day (Akk1) or 82 mg/kg/day (Akk2) for another 4 weeks. Gut microbiota was analyzed via metagenomic sequencing, and gene expression was evaluated through transcriptomics.

**Results:**

LPS significantly altered gut microbiota, reducing diversity and increasing pathogenic genera like Lachnoclostridium. *Akkermansia muciniphila* supplementation, particularly at higher doses, partially restored gut microbiota by increasing beneficial genera such as Muribaculum. Transcriptomics showed that LPS induced immune and inflammatory responses, while *Akkermansia muciniphila* reduced these effects by modulating pathways like TNF and NF-kappa B signaling.

**Conclusion:**

*Akkermansia muciniphila* mitigates LPS-induced gut dysfunction by restoring microbiota balance and modulating immune responses, highlighting its potential as a therapeutic agent for gut health.

## 1 Introduction

Mild intestinal dysfunction, often referred to as slight intestinal dysfunction, may not manifest significant symptoms immediately but can potentially affect overall health if it persists over the long term (Camilleri et al., 2012; Forlano et al., 2022; Brandt et al., 2022). This condition can subtly but significantly impact digestive comfort, nutrient absorption, gut microbiota balance, immune response, weight, metabolism, and pose other long-term health risks (Miner-Williams and Moughan, 2016; Shi et al., 2023). Various factors, such as poor dietary habits, lifestyle choices, and environmental influences, can contribute to mild intestinal dysfunction (Shi et al., 2023; McKenna et al., 2022). Interventions, including dietary improvements and supplementation with probiotics and prebiotics, have shown promise in restoring gut microbiota balance and improving intestinal health (Dargenio et al., 2023; Camara-Lemarroy et al., 2018). However, more research is needed to elucidate the relationship between gut microbiota and intestinal health and to develop effective intervention strategies.

Probiotic supplementation has been shown to have several beneficial effects on mild intestinal dysfunction by improving gut microbiota balance and regulating immune responses (Suez et al., 2019; Dale et al., 2019). Beyond traditional probiotics like lactobacilli and bifidobacteria, new probiotics continue to be discovered and studied (Abouelela and Helmy, 2024; Al-Fakhrany and Elekhnawy, 2024). *Akkermansia muciniphila*, first isolated and described in 2004, has emerged as a notable member of the gut microbiota due to its beneficial roles in human health (Derrien et al., 2004). *Akkermansia muciniphila* specializes in degrading mucins, which are glycoproteins forming a protective layer in the gut, and its activities are associated with various health benefits, including regulation of immune responses, metabolic processes, and protection against inflammatory and neurodegenerative disorders (Mruk-Mazurkiewicz et al., 2024; Zhang et al., 2019; Cani et al., 2022). The health-promoting effects of *Akkermansia muciniphila* are largely attributed to its production of short-chain fatty acids (SCFAs) like acetate and propionate, which have anti-inflammatory properties and contribute to metabolic regulation, suggesting roles in weight management and immune modulation (Zhang and Wang, 2023; Shen et al., 2023).

To further explore the role of probiotics in mild intestinal dysfunction, we utilized Lipopolysaccharide (LPS) to construct animal models of mild intestinal dysfunction. LPS, a component of the outer membrane of gram-negative bacteria, can induce intestinal mucosal damage by interacting with the host’s immune system, disrupting the intestinal barrier, and activating inflammatory pathways. The interaction of LPS with the intestinal epithelium increases gut permeability, allowing harmful substances and pathogens to enter systemic circulation, potentially leading to “bacterial translocation” and triggering systemic inflammatory responses.

In this study, we hypothesized that *Akkermansia muciniphila* could exert therapeutic effects on intestinal dysfunction induced by LPS. We designed a mouse model of intestinal disorder induced by LPS feeding and administered different doses of *Akkermansia* probiotic formulations during the recovery period. Our goal was to explore the effective role of *Akkermansia* as a probiotic in this intestinal disorder model. By understanding the interactions between *Akkermansia muciniphila* and the gut microbiota in the context of LPS-induced intestinal dysfunction, we aim to contribute to the development of probiotic-based therapies for maintaining and restoring intestinal health.

## 2 Methods

### 2.1 Animals and diets

This study was approved by the Shenzhen Institute for Drug Control and Shenzhen InnoStar Institute of Biomedical Safety Evaluation and Research Co., Ltd., and was conducted in accordance with the National Research Council’s Guide for the Care and Use of Laboratory Animals. Thirty-eight 6-week-old C57BL/6 mice (equal numbers of males and females) were randomly divided into two groups after 1 week of acclimation. The CON group (*n* = 19) received a basic diet, while the LPS group (*n* = 19) received a diet supplemented with LPS (300 μg/kg/day) for 4 weeks. LPS (Solarbio, batch number: 2101034, purity ≥98%) was purchased in powder form and dissolved in sterile saline before being administered orally via gavage once daily. Phenotypic data were collected weekly.

After 4 weeks of LPS exposure, stools and colon tissues were collected from half of the CON (*n* = 11) and LPS (*n* = 11) groups following euthanasia for further analysis. Euthanasia was performed using an overdose of carbon dioxide (CO_2_) in a controlled chamber, following guidelines to ensure minimal discomfort. The flow rate of CO_2_ was set to displace 30–70% of the chamber volume per minute, as recommended by the National Research Council’s Guide for the Care and Use of Laboratory Animals.

The remaining mice from the CON and LPS groups were then fed *Akkermansia* for an additional 4 weeks. *Akkermansia muciniphila* was obtained in the form of a commercially available probiotic capsule (Pendulum Akkermansia, Pendulum Life, United States). The contents of each capsule, which were in powder form, were dissolved in sterile saline and administered orally via gavage at doses of 41 mg/kg/day (Akk1 group) or 82 mg/kg/day (Akk2 group) once daily. These mice were subdivided into new groups based on the dose of *Akkermansia*: CON-Akk1 (Akk = 41 mg/kg/day, *n* = 4), CON-Akk2 (Akk = 82 mg/kg/day, *n* = 4), LPS-Akk1 (Akk = 41 mg/kg/day, *n* = 4), and LPS-Akk2 (Akk = 82 mg/kg/day, *n* = 4). After the additional 4 weeks, stools and colon tissues were collected post-euthanasia for further analysis.

### 2.2 DNA extraction and RNA isolation

Total DNA was extracted from stool samples using the CTAB/SDS method. The DNA was resuspended in 50 μL of DES (DNase/Pyrogen-Free Water) and quantified using a Qubit 3.0 Fluorometer. Total RNA from colon tissues was extracted using the QIAGEN RNeasy Protect Animal Tissue Kit (QIAGEN, Germany). RNA degradation and contamination were monitored using 1% agarose gels. RNA purity was assessed with a NanoPhotometer spectrophotometer (IMPLEN, CA, United States), and RNA concentration was measured using a Qubit RNA Assay Kit with a Qubit 2.0 Fluorometer (Life Technologies, CA, United States). RNA integrity was evaluated using the RNA Nano 6000 Assay Kit on an Agilent Bioanalyzer 2100 system (Agilent Technologies, CA, United States).

### 2.3 Library preparation, metagenome sequencing, and data analysis of gut microbiome

The construction of a metagenomic library primarily utilizes conventional small DNA fragment library technology. Qualified DNA samples are randomly fragmented into approximately 350 bp fragments using Covaris. The entire library preparation process includes end repair, A-tailing, sequencing adapter ligation, purification, and PCR amplification. After library construction, initial quantification is performed using Qubit 2.0, and the library is diluted to 2 ng/μL. The insert size of the library is then detected using the Agilent 2100. Once the insert size meets the expected specifications, the effective concentration of the library is quantified using the q-PCR method, ensuring a library effective concentration of greater than 3 nM. Following quality control, different libraries are pooled based on their effective concentration and the required volume of target sequencing data. The pooled libraries are then subjected to Illumina sequencing using the PE150 sequencing strategy.

### 2.4 Library preparation, sequencing and transcriptome analysis of intestinal tissues

A total of 1.5 μg RNA per sample was used as input material for RNA sample preparations. Sequencing libraries were generated using the NEBNext UltraTM Directional RNA Library Prep Kit for Illumina (NEB, United States) following the manufacturer’s recommendations. Briefly, mRNA was purified from total RNA using poly-T oligo-attached magnetic beads. Fragmentation was carried out using divalent cations under elevated temperature in NEBNext First Strand Synthesis Reaction Buffer (5X). First strand cDNA was synthesized using random hexamer primers and M-MuLV Reverse Transcriptase (RNase H-). Second strand cDNA synthesis was subsequently performed using DNA Polymerase I and RNase H. In the reaction buffer, dNTPs with dTTP were replaced by dUTP. Remaining overhangs were converted into blunt ends via exonuclease/polymerase activities. After adenylation of the 3′ ends of DNA fragments, NEBNext Adaptors with hairpin loop structures were ligated to prepare for hybridization. To select cDNA fragments of the right length, the library fragments were purified with the AMPure XP system (Beckman Coulter, Beverly, United States). Then, 3 μL of USER Enzyme (NEB, USA) was used with size-selected, adaptor-ligated cDNA at 37°C for 15 min followed by 5 min at 95°C before PCR. PCR was then performed with Phusion High-Fidelity DNA Polymerase, Universal PCR Primers, and Index (X) Primer. Finally, products were purified with the AMPure XP system and library quality was assessed on the Agilent Bioanalyzer 2100 system.

From these libraries, 150-bp paired-end and strand-specific sequence reads were produced with the Illumina HiSeq X Ten. Tophat2 (v2.1.0) was used for read mapping to the *Sus scrofa* genome reference (v11.1) (Ramya et al., 2021). Gene expression values were normalized using the number of metric fragments per kilobase of exon region per million mapped reads (FPKM) with Cufflinks (version 2.2.1) (Trapnell et al., 2012). Differentially expressed genes (DEGs) were identified with an adjusted *p*-value cutoff of 0.05 and an absolute fold-change of 2. GO enrichment of DEGs was performed using a hypergeometric test based on the *Sus scrofa* background, and the results were visualized using the GO plot package in R (Xu et al., 2021). A multivariate linear fitting model was used to investigate the associations among SCFAs, gut microbes, and DEGs using the R package lm. The formula in our model is as follows:


M~aS+bF+c.

Where *M* is the concentration of SCFAs for all samples, *S* is the abundance of microbes for all samples, *F* is gene expression values of DEGs for all samples, *a* and *b* are coefficients, *c* is the intercept. The evaluation thresholds for models were adjusted *R*^2^ > =0.8 and *p* value < 0.05.

### 2.5 Biochemical analysis

The levels of triglycerides (TG), alanine aminotransferase (ALT), and aspartate aminotransferase (AST) were measured from serum samples collected at the end of the experiment. Blood samples were collected via cardiac puncture, allowed to clot at room temperature for 30 min, and centrifuged at 3,000 × g for 10 min at 4°C to obtain serum. TG levels were quantified using an enzymatic colorimetric method (Shenzhen Mindray Bio-Medical Electronics, 141721011). ALT and AST activities were measured using an automated biochemical analyzer (Shenzhen Mindray Bio-Medical Electronics Co., Ltd., BS-800 M) with specific assay kits (Shenzhen Mindray Bio-Medical Electronics Co., Ltd.; ALT, 140122006; AST, 140222007). All assays were performed in triplicate according to the manufacturer’s instructions to ensure accuracy and reliability.

### 2.6 Statistical analysis

Experimental data were expressed as mean ± standard deviation (Mean ± SD). Differences between groups were evaluated using one-way ANOVA, followed by Tukey’s HSD test for post-hoc analysis. For data that did not meet the normality assumption, non-parametric tests (Mann–Whitney U test or Kruskal-Wallis test) were used. For transcriptomic data, differentially expressed genes (DEGs) were identified using DESeq2 software with a threshold of adjusted *p*-value < 0.05 and an absolute fold change >2 or <0.5. Gene Ontology (GO) and KEGG pathway enrichment analyses were performed using hypergeometric tests and visualized with the GOplot package in R.

In gut microbiota diversity analysis, alpha diversity was calculated using the Shannon diversity index, and differences between groups were tested with the Wilcoxon rank-sum test. Microbial community composition differences were assessed via Bray-Curtis distance-based Principal Coordinate Analysis (PCoA), and statistical significance between groups was tested using PERMANOVA. Correlations between gut microbiota and key immune-related genes were analyzed using Pearson correlation analysis, and protein–protein interaction (PPI) networks were constructed in R to visualize significant associations between genes and microbial species. All statistical analyses were performed using GraphPad Prism 9 and R (version 4.2.2), with statistical significance set at *p* < 0.05.

## 3 Results

### 3.1 Impact of lipopolysaccharide and *Akkermansia muciniphila* on metabolic and immunological parameters

**Figure 1:**
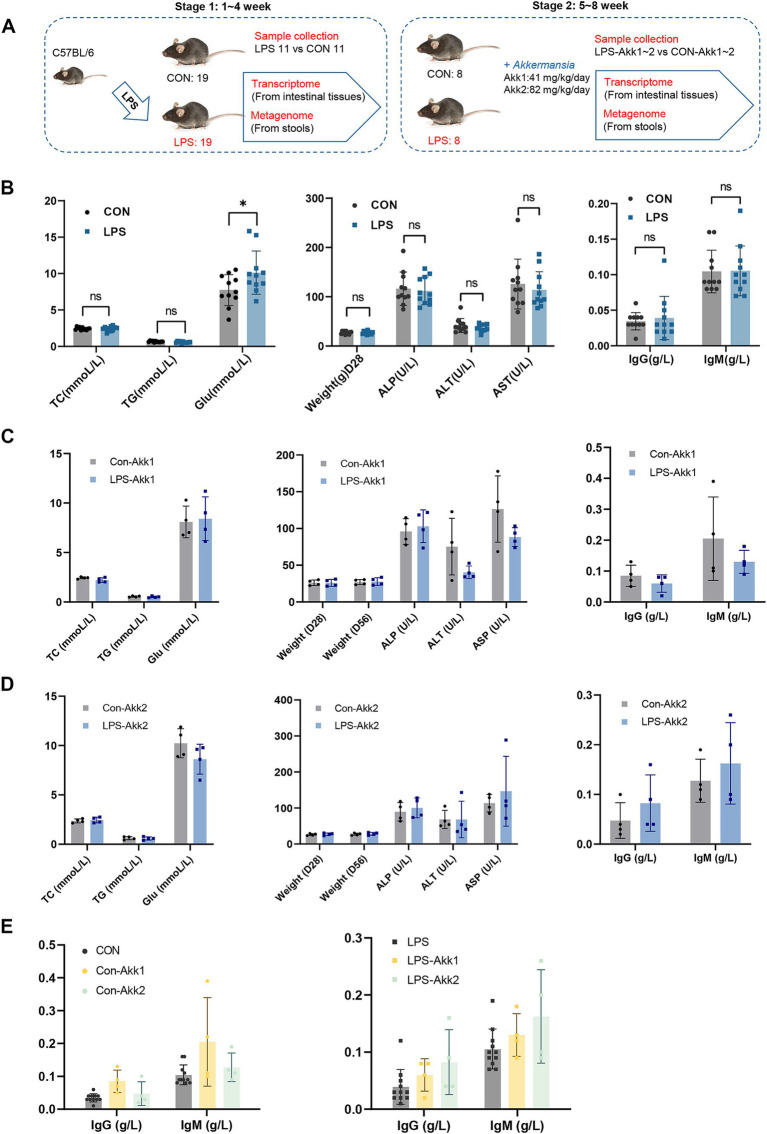
Construction of animal model and phenotypic comparison. **(A)** Experimental Design: C57BL/6 mice were divided into two groups: control (CON, *n* = 19) and LPS-treated (LPS, *n* = 19). Saline solution or LPS (300 μg/kg/day) was administered for 4 weeks. Samples were collected for transcriptomic and metagenomic analysis. In the second stage, remaining mice were divided and treated with *Akkermansia muciniphila* at doses of 41 mg/kg/day (Akk1) and 82 mg/kg/day (Akk2) for another 4 weeks, with further sample collections. **(B)** Comparison of metabolic and immunological parameters between control (CON) and LPS-treated (LPS) mice, including total cholesterol (TC), triglycerides (TG), body weight (Weight), ALP (Alkaline Phosphatase), alanine aminotransferase (ALT), aspartate aminotransferase (AST), immunoglobulin G (IgG), and immunoglobulin M (IgM). **(C)** Comparison of metabolic and immunological parameters between control (CON + Akk) and LPS-treated (LPS + Akk) mice receiving 41 mg/kg/day or **(D)** 82 mg/kg/day of *Akkermansia muciniphila*. **(E)** Comparison of metabolic and immunological parameters between control (CON + Akk2) and LPS-treated (LPS + Akk2) mice receiving 82 mg/kg/day of *Akkermansia muciniphila*. **(E)** A comparison of the levels of immunoglobulin G (IgG) and immunoglobulin M (IgM) in serum samples between the control group (CON) and mice treated with LPS after supplementation with *Akkermansia muciniphila* at two distinct dosages, namely Akk1 and Akk2.

The experimental design involved two main stages over 8 weeks (Figure 1A). In the first stage (weeks 1–4), 38 C57BL/6 mice were divided into control (CON, *n* = 19) and LPS-treated (LPS, *n* = 19) groups. LPS was administered at 300 μg/kg/day to induce mild intestinal dysfunction. After 4 weeks, samples from half of mice in CON and LPS groups were collected for transcriptomic and metagenomic analysis. In the second stage (weeks 5–8), remaining mice were further divided and treated with *Akkermansia muciniphila* at doses of 41 mg/kg/day (Akk1) and 82 mg/kg/day (Akk2). The phenotypic data collected during both stages provide insights into the effects of LPS and *Akkermansia muciniphila* on the mice.

During the first stage, LPS administration induced a significant increase in blood glucose levels (*p < 0.05) compared to controls (Figure 1B). However, there were no significant differences between the CON and LPS groups in total cholesterol (TC), triglycerides (TG), body weight, ALP (Alkaline Phosphatase), alanine aminotransferase (ALT), aspartate aminotransferase (AST), immunoglobulin G (IgG), and immunoglobulin M (IgM) levels. In the second stage, the effects of *Akkermansia muciniphila* supplementation on LPS-treated mice were evaluated. For mice receiving 41 mg/kg/day (Akk1), there were no significant differences in TC, TG, glucose levels, body weight, ALT, AST, IgG, and IgM between the LPS + Akk1 and CON + Akk1 groups (Figure 1C). Similarly, for mice receiving 82 mg/kg/day (Akk2), no significant differences were observed in these parameters between the LPS + Akk2 and CON + Akk2 groups (Figure 1D). A comprehensive comparison of immunoglobulin levels across all experimental groups revealed no significant changes in IgG or IgM levels due to LPS administration or *Akkermansia* supplementation (Figure 1E).

These results indicate that while LPS administration induces a significant increase in blood glucose levels, other metabolic and immunological parameters remain unaffected. Moreover, *Akkermansia muciniphila* supplementation at the tested doses did not significantly alter these parameters, suggesting a need for further analysis of the gut microbiome and gene expression profiles to elucidate potential underlying mechanisms.

### 3.2 Transcriptome analysis of intestinal tissues

**Figure 2:**
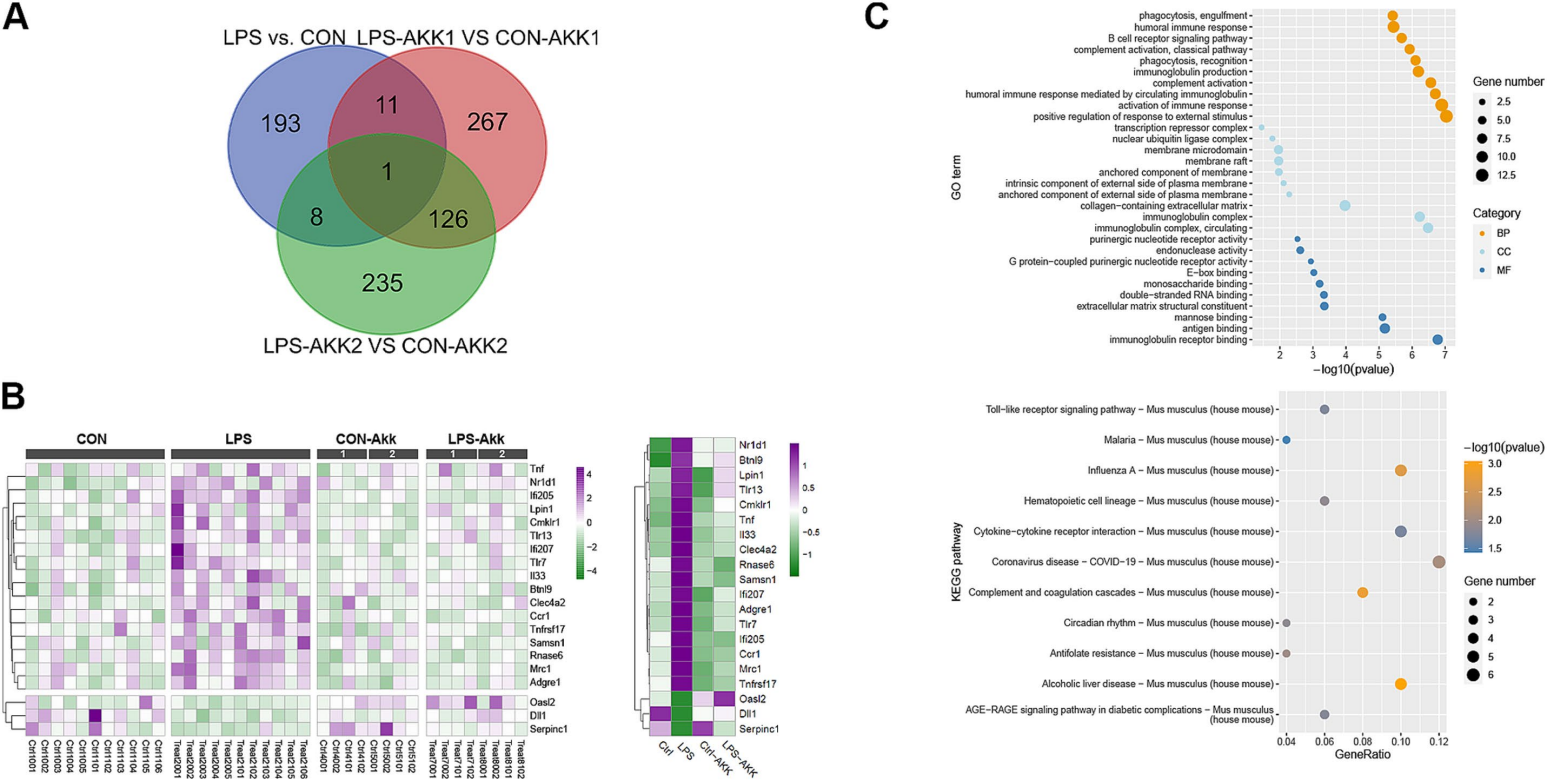
Function analysis of the immune DEGs between LPS and CON groups. **(A)** Venn diagram showing the overlap of differentially expressed genes (DEGs) among the comparisons of LPS vs. CON, LPS-Akk1 vs. CON-Akk1, and LPS-Akk2 vs. CON-Akk2 groups. **(B)** GO enrichment and KEGG pathway analysis of DEGs. The top panel displays the GO terms and the bottom panel shows KEGG pathway analysis. **(C)** Heatmap illustrating the expression levels of key immune-related DEGs across CON, LPS, CON-Akk, and LPS-Akk groups.

RNA extracted from intestinal tissues was subjected to transcriptome sequencing to assess the impact of LPS and *Akkermansia muciniphila* on gene expression. Principal Component Analysis (PCA) of the transcriptome data revealed distinct differences in gene expression profiles between the LPS and CON groups, indicating significant changes induced by LPS administration. These differences in gene expression profiles were notably reduced after feeding with *Akkermansia muciniphila*, as shown by the overlapping clusters for the LPS-Akk1, LPS-Akk2, CON-Akk1, and CON-Akk2 groups (Figure 2A). The analysis of differentially expressed genes (DEGs) further highlighted the impact of LPS and *Akkermansia* supplementation. Volcano plots illustrated a high number of DEGs between the LPS and CON groups (adj *p*-value < 0.05, fold change >2 or <0.5). In contrast, the number of DEGs was significantly reduced in the comparisons between LPS-Akk1 vs. CON-Akk1 and LPS-Akk2 vs. CON-Akk2 groups, suggesting that *Akkermansia muciniphila* mitigates the changes in gene expression induced by LPS (Figure 2B).

**Figure 3:**
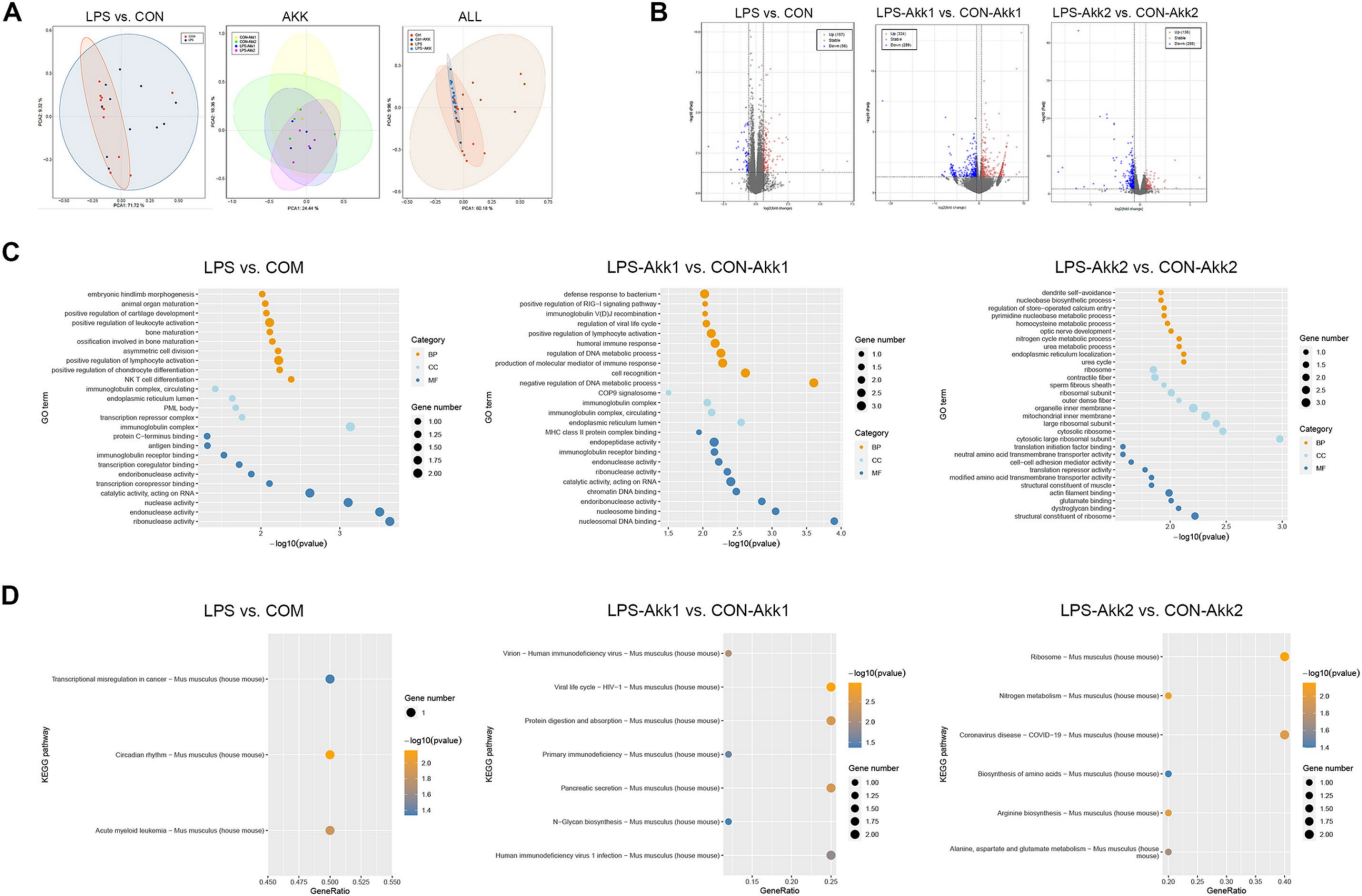
Transcriptome analysis. **(A)** PCA plots showing the distinct clustering of intestinal transcriptome profiles between the LPS and control (CON) groups. **(B)** Volcano plots illustrating the DEGs between LPS vs. CON, LPS-Akk1 vs. CON-Akk1, and LPS-Akk2 vs. CON-Akk2 groups. DEGs were identified based on an adjusted *p*-value < 0.05 and a fold change >2 or <0.5. Red dots represent upregulated genes, blue dots represent downregulated genes, and black dots indicate genes with no significant change. **(C)** GO enrichment analysis of DEGs reveals significant involvement of immune-related functions and pathways in the LPS vs. CON comparison. BP (Biological Process), CC (Cellular Component), and MF (Molecular Function). **(D)** KEGG pathway analysis shows enrichment of immune function and inflammation-related pathways.

Gene Ontology (GO) enrichment analysis of DEGs between the LPS and CON groups indicated significant involvement in immune-related functions and pathways. Notably, DEGs were enriched in terms such as “immunoglobulin receptor binding” and “antigen binding,” which are indicative of an immune response triggered by LPS administration. Furthermore, pathways associated with inflammation and immune response, such as “cytokine activity” and “chemokine receptor binding,” were also significantly enriched in the LPS group. Following *Akkermansia* supplementation, especially at the higher dose (Akk2), these immune-related pathways were substantially less prominent in the LPS-Akk vs. CON-Akk comparisons. In the LPS-Akk2 group, the enrichment of immune response-related GO terms was markedly reduced, suggesting a modulation of the immune response by *Akkermansia muciniphila*. The terms associated with “antigen processing and presentation” and “leukocyte migration” were particularly decreased, indicating a potential normalization of the immune function (Figure 2C). KEGG pathway analysis supported the findings of the GO enrichment analysis. In the LPS group, several pathways related to immune and inflammatory responses were significantly enriched, including “Toll-like receptor signaling pathway,” “Cytokine-cytokine receptor interaction,” and “Complement and coagulation cascades” (Figure 3B). These pathways are critical mediators of the inflammatory response triggered by LPS administration. Additionally, pathways associated with disease and infection, such as “Influenza A” and “Coronavirus disease-COVID-19,” were also significantly enriched, highlighting the broad immune activation induced by LPS (Figure 2C).

In summary, transcriptome analysis revealed that LPS administration significantly alters intestinal gene expression, primarily affecting immune-related pathways and inflammatory responses. *Akkermansia muciniphila* supplementation, particularly at higher doses, appeared to mitigate these changes, reducing the number of DEGs and the prominence of immune-related and inflammatory pathways, indicating its potential therapeutic role in modulating LPS-induced intestinal dysfunction.

### 3.3 Functional analysis of immune-related DEGs between LPS and CON groups

To further investigate the functional impact of differentially expressed genes (DEGs) related to the immune response, we performed a series of analyses comparing LPS-treated and control (CON) groups, as well as the effects of *Akkermansia muciniphila* supplementation.

A Venn diagram was used to identify shared and unique DEGs across the LPS vs. CON, LPS-Akk1 vs. CON-Akk1, and LPS-Akk2 vs. CON-Akk2 comparisons. The analysis revealed a total of 193 unique DEGs in the LPS vs. CON comparison, 267 unique DEGs in the LPS-Akk1 vs. CON-Akk1 comparison, and 235 unique DEGs in the LPS-Akk2 vs. CON-Akk2 comparison (Figure 3A). There was a considerable overlap, with some DEGs common to all three groups, indicating a core set of genes consistently affected by LPS treatment and partially modulated by *Akkermansia* supplementation.

Gene Ontology (GO) enrichment analysis of the DEGs between LPS and CON groups highlighted significant involvement in immune-related biological processes (BP), such as “phagocytosis, engulfment,” “B cell humoral immune response,” and “complement activation.” Molecular functions (MF) included “immunoglobulin receptor binding” and “G protein-coupled purinergic nucleotide receptor activity,” while cellular components (CC) involved “anchored component of external side of plasma membrane” (Figure 3B). These findings suggest that LPS administration elicits a robust immune response. Following *Akkermansia* supplementation, particularly at the higher dose (Akk2), the GO enrichment analysis showed a marked reduction in the prominence of immune-related pathways. In the LPS-Akk2 vs. CON-Akk2 comparison, immune-related terms such as “humoral immune response mediated by circulating immunoglobulin” and “phagocytosis, recognition” were less enriched, indicating a dampening of the immune response. KEGG pathway analysis further supported the GO enrichment findings, showing significant enrichment in immune and inflammatory pathways in the LPS vs. CON comparison (Figure 3D). Pathways such as “Toll-like receptor signaling,” “Cytokine-cytokine receptor interaction,” and “Complement and coagulation cascades” were prominently upregulated in response to LPS (Figure 3B). Notably, pathways associated with viral infections, such as “Influenza A” and “Coronavirus disease-COVID-19,” were also enriched, reflecting the broad immune activation induced by LPS. In the *Akkermansia*-treated groups, particularly LPS-Akk2, these pathways were significantly less enriched. The “Toll-like receptor signaling pathway” and “Cytokine-cytokine receptor interaction” showed reduced activity, suggesting that *Akkermansia muciniphila* supplementation mitigates the inflammatory response triggered by LPS. A heatmap illustrated the expression levels of key immune-related DEGs across all experimental groups (Figure 3C). Genes such as Tnf, Nr1d1, and Ifi205 were significantly upregulated in the LPS group compared to the CON group. *Akkermansia* supplementation, particularly at the higher dose (Akk2), led to a normalization of these gene expression levels. For instance, the expression of Tnf and Nr1d1 was markedly reduced in the LPS-Akk2 group, approaching levels observed in the CON group.

### 3.4 Functional analysis of immune-related differentially expressed genes

**Figure 4:**
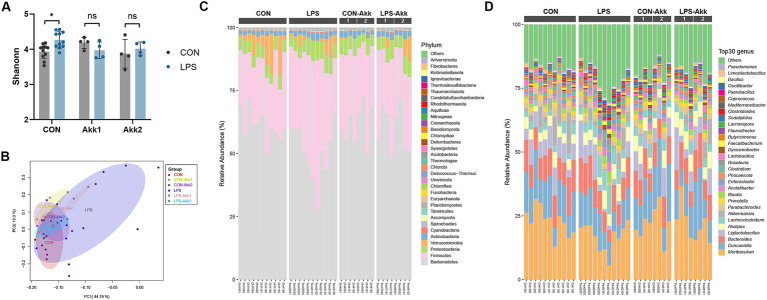
Effects of LPS treatment and *Akkermansia* supplementation on gut microbiota composition. **(A)** Shannon diversity index of microbial communities in the CON, LPS, CON-Akk1, CON-Akk2, LPS-Akk1, and LPS-Akk2 groups. **(B)** PCoA based on Bray–Curtis dissimilarity, showing distinct clustering of microbial communities among the different treatment groups. **(C)** Relative abundance of microbial phyla across different treatment groups. **(D)** Relative abundance of the top 30 microbial genera across different treatment groups.

The impact of LPS administration and *Akkermansia muciniphila* supplementation on the intestinal microbiota was assessed through metagenome analysis. Shannon diversity index analysis revealed a significant increase in microbial diversity in the LPS group compared to the CON group (*p < 0.05) (Figure 4A). However, no significant differences in alpha diversity were observed between the CON and LPS groups following *Akkermansia* supplementation at either dose (Akk1 and Akk2), suggesting that *Akkermansia* may help maintain microbial diversity in the presence of LPS-induced dysbiosis. Principal Coordinate Analysis (PCoA) based on Bray-Curtis distances demonstrated distinct clustering of the microbial communities (Figure 4B). The LPS group formed a separate cluster from the CON group, indicating significant changes in microbial composition due to LPS treatment. *Akkermansia* supplementation resulted in a shift in microbial communities, with the LPS-Akk1 and LPS-Akk2 groups showing a microbial composition more similar to the CON group, highlighting the modulatory effect of *Akkermansia* on the gut microbiota. The relative abundance of microbial phyla showed significant changes in response to LPS treatment (Figure 4C). Specifically, the LPS group exhibited an increased relative abundance of Bacillota and a decreased abundance of Bacteroidota compared to the CON group. Supplementation with *Akkermansia*, especially at the higher dose (Akk2), partially restored the Bacteroidota levels and reduced the Bacillota abundance, indicating a normalization of the gut microbiota.

At the genus level (Figure 4D), LPS administration led to significant changes in the abundance of various genera. Specifically, the LPS group exhibited an increase in the relative abundance of potentially pathogenic genera such as *Lachnoclostridium* and *Ligilactobacillus*, and a decrease in beneficial genera such as *Muribaculum* compared to the CON group. Supplementation with *Akkermansia*, particularly at the higher dose (Akk2), partially restored the levels of beneficial genera and reduced the abundance of pathogenic genera, indicating a normalization of the gut microbiota. Metagenome analysis revealed that LPS administration significantly disrupts the gut microbiota, reducing microbial diversity and altering the composition at both phylum and genus levels. *Akkermansia muciniphila* supplementation mitigates these effects, maintaining microbial diversity and restoring a more balanced and beneficial microbial community. These findings underscore the potential of *Akkermansia muciniphila* to counteract LPS-induced dysbiosis and promote gut health.

### 3.5 Correlation analysis reveals significant interactions between gut microbiota and key immune-related genes

**Figure 5:**
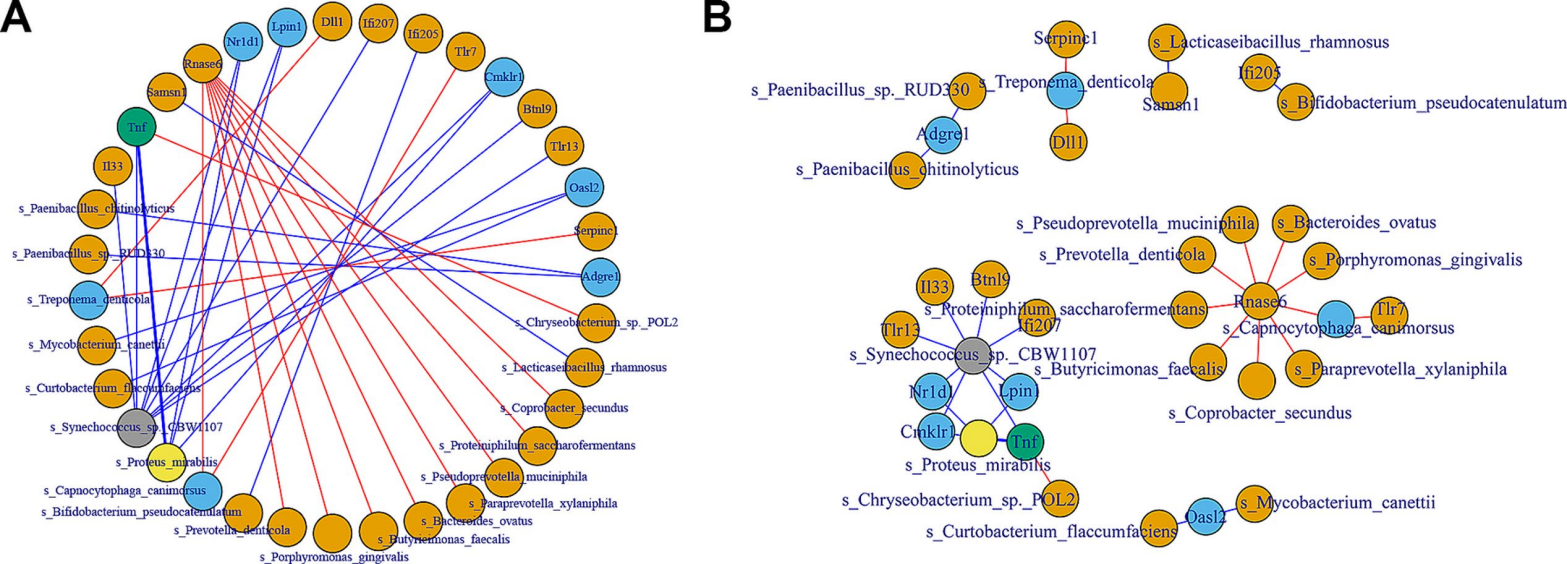
Correlation analysis between key immune DEGs and gut microbiome. **(A)** The PPI network illustrates the interactions between selected immune-related genes and gut microbiota. The network nodes represent genes and microbial species, with edges indicating significant interactions. Blue lines represent negative correlations, while red lines represent positive correlations. **(B)** The Pearson correlation network shows the strength and direction of the relationships between the 20 immune-related genes and the gut microbiota. Positive correlations are represented by orange lines, and negative correlations are shown with blue lines.

To investigate the relationship between key immune-related genes (DEGs) and gut microbiota, we performed correlation analyses using both Pearson correlation and protein–protein interaction (PPI) networks. The PPI network revealed significant interactions between several immune genes and microbial species (Figure 5A). Notably, genes such as Tlr1, Il33, and Btnl9 were central in the network, indicating their potential role in modulating immune responses through interactions with gut microbiota. For instance, Tlr1 showed numerous positive correlations with microbial species such as *Synechococcus* sp., while Il33 displayed both positive and negative correlations, indicating its complex regulatory role. The Pearson correlation analysis (Figure 5B) further identified specific microbial taxa significantly correlated with the expression of immune genes. Positive correlations were observed between Tlr1 and *Synechococcus* sp. (CBW1107), while negative correlations were found between Il33 and *Actinomycetota* sp. These findings suggest that certain gut microbiota species are closely linked to immune gene regulation, with potential implications for understanding the microbiome-immune system interactions.

These analyses highlight the intricate relationships between gut microbiota and immune-related genes, providing insights into how microbial communities may influence host immune responses. The identification of specific microbial species correlated with immune gene expression underscores the importance of the gut microbiome in maintaining immune homeostasis and potential targets for therapeutic interventions.

## 4 Discussion

Lipopolysaccharide (LPS), also known as endotoxin, is a component of the outer membrane of Gram-negative bacteria that can significantly impact intestinal function when it crosses the intestinal barrier (Ghosh et al., 2020; An et al., 2022; Anhe et al., 2021). LPS can induce intestinal mucosal damage by interacting with the host’s immune system, leading to disruption of the intestinal barrier and subsequent activation of inflammatory pathways (An et al., 2022; Amoroso et al., 2020). The interaction of LPS with the intestinal epithelium increases gut permeability, allowing harmful substances and pathogens to enter systemic circulation (Ghosh et al., 2020; Bein et al., 2017; Allam-Ndoul et al., 2020). This condition, known as “bacterial translocation,” can trigger a systemic inflammatory response. The binding of LPS to Toll-like receptor 4 (TLR4) on immune cells initiates a signaling cascade that activates the transcription of various inflammatory cytokines, such as interleukin-1 beta (IL-1β), interleukin-6 (IL-6), and tumor necrosis factor-alpha (TNF-α), contributing to intestinal inflammation (Ciesielska et al., 2021; Coutinho-Wolino et al., 2022).

Our study investigated the potential therapeutic effects of *Akkermansia muciniphila* on LPS-induced mild intestinal dysfunction, using transcriptomic and metagenomic analyses. The findings align with previous research demonstrating the detrimental effects of LPS on gut microbiota and its role in inducing inflammatory pathways. For instance, Cani et al. reported that LPS induces metabolic endotoxemia, leading to systemic inflammation and insulin resistance (Cani et al., 2007). Consistently, our study showed that LPS administration led to significant alterations in gut microbiota composition, increased inflammatory responses, and disrupted intestinal gene expression.

*Akkermansia muciniphila* has been extensively highlighted in the literature for its beneficial roles in gut health. Everard et al. demonstrated that *Akkermansia muciniphila* supplementation improved gut barrier function and reduced metabolic endotoxemia in obese mice (Everard et al., 2013). Similarly, our study showed that supplementation with *Akkermansia muciniphila*, particularly at higher doses (82 mg/kg/day), significantly alleviated LPS-induced dysbiosis and immune activation. These findings not only corroborate previous studies but also extend the understanding of *Akkermansia muciniphila*’s effects by focusing specifically on an LPS-induced model of mild intestinal dysfunction, which closely resembles subclinical inflammation observed in various chronic conditions (Candelli et al., 2021; Ji et al., 2019). This model allowed us to evaluate the therapeutic potential of *Akkermansia muciniphila* in mitigating mild, yet potentially harmful, gut dysfunction, providing new insights into its role in intestinal health.

LPS treatment induced profound changes in gut microbiota composition, as evidenced by increased relative abundances of Firmicutes and decreased abundances of Bacteroidetes and Verrucomicrobia. These phylum-level changes are consistent with previous reports linking LPS-induced dysbiosis to inflammation and metabolic disturbances. Notably, Verrucomicrobia, which includes *Akkermansia muciniphila*, showed a reduced relative abundance in LPS-treated animals, likely reflecting the depletion of mucin-degrading bacteria under inflammatory conditions. This reduction was partially restored following *Akkermansia muciniphila* supplementation, particularly at the higher dose, suggesting a direct impact of Akkermansia on the gut ecosystem.

At the genus level, LPS treatment increased the abundance of potentially pathogenic genera such as Lachnoclostridium and Ligilactobacillus, which are associated with inflammation and gut barrier dysfunction. Conversely, beneficial genera such as Muribaculum, which play roles in mucin degradation and short-chain fatty acid (SCFA) production, were significantly reduced. Supplementation with *Akkermansia muciniphila* reversed these trends by increasing the abundance of Muribaculum and reducing the abundance of Lachnoclostridium, contributing to the normalization of the gut microbiota. The observed restoration of gut microbiota and reduction of inflammation underscore *Akkermansia muciniphila*’s potential as a therapeutic agent beyond severe gut conditions, suggesting broader applications in preventive healthcare (Luo et al., 2021).

The transcriptomic data revealed that LPS treatment upregulated immune and inflammatory pathways, including cytokine-cytokine receptor interaction, Toll-like receptor signaling, and NF-kappa B signaling, which are key mediators of intestinal inflammation. These findings are consistent with earlier studies linking LPS to immune activation and gut barrier dysfunction (Chen et al., 2017; Webster and Vucic, 2020). Supplementation with *Akkermansia muciniphila*, particularly at higher doses, significantly downregulated these pathways, suggesting an anti-inflammatory effect. Notably, the expression of pro-inflammatory markers such as Tnf, Il6, and Cd14 showed trends of normalization in the Akkermansia-treated groups, aligning with the reduction in immune-related pathways observed in KEGG analysis. In addition, the enrichment of pathways related to antigen presentation and leukocyte migration was reduced in Akkermansia-treated animals, indicating a potential restoration of immune homeostasis. These findings highlight the ability of *Akkermansia muciniphila* to modulate molecular pathways associated with inflammation, maintaining immune homeostasis and providing a mechanistic basis for its therapeutic effects. The reduction of key inflammatory pathways, such as TNF signaling and NF-kappa B signaling pathways, suggests that *Akkermansia muciniphila* could mitigate chronic inflammatory conditions through molecular modulation (Webster and Vucic, 2020).

Microbial diversity, a critical indicator of gut health, was significantly reduced in the LPS group. Supplementation with *Akkermansia muciniphila* partially restored alpha diversity, as shown by the Shannon index, and shifted the microbial community composition closer to that of the control group. These findings suggest that *Akkermansia muciniphila* not only mitigates the effects of LPS-induced dysbiosis but also promotes a more balanced and resilient microbial ecosystem, which is crucial for maintaining intestinal homeostasis.

In conclusion, our study demonstrates that *Akkermansia muciniphila* supplementation effectively mitigates LPS-induced intestinal dysfunction by restoring gut microbiota balance, reducing inflammation, and modulating immune-related pathways. The detailed transcriptome analysis provided new insights into the specific immune pathways and genes modulated by *Akkermansia muciniphila*, highlighting its potential in maintaining immune homeostasis. These findings contribute to the growing body of evidence supporting the use of *Akkermansia muciniphila* as a therapeutic agent for gut health, with potential applications in both subclinical and chronic inflammatory conditions.

## Data Availability

The data presented in this study are deposited in the NCBI Sequence Read Archive (SRA) repository, accession number PRJNA1225863.
